# Assessment of the Levels of Level of Biomarkers of Bone Matrix Glycoproteins and Inflammatory Cytokines from Saudi Parkinson Patients

**DOI:** 10.1155/2019/2690205

**Published:** 2019-05-08

**Authors:** Aziza Alrafiah, Ebtisam Al-Ofi, Mona Talib Obaid, Nimah Alsomali

**Affiliations:** ^1^Department of Medical Laboratory Technology, Faculty of Applied Medical Sciences, King AbdulAziz University, Jeddah, Saudi Arabia; ^2^Department of Physiology, Faculty of Medicine, King Abdulaziz University, Jeddah, Saudi Arabia; ^3^National Neuroscience Institute, King Fahad Medical City, Riyadh, Saudi Arabia

## Abstract

*Background. *Parkinson's disease (PD) is the second most commonly neurodegenerative disease after Alzheimer's disease which occurs to nearly 1% of the population > 50 years old. Inflammatory and bone biomarkers have both become valuable tools for PD diagnosis and prognosis. However, no studies have examined these markers in Saudi patients diagnosed with PD.* Objectives.* To assess the biomarkers and proinflammatory cytokines from blood with PD in serum.* Methods.* In our study, we included 26 patients with PD and 24 controls. Blood samples were withdrawn from subjects with PD and their matched controls. Biomarkers multiplex assay from Milliplex was used to assess the levels of IL-1B, IL-6, TNF-*α*, osteoprotegerin (OPG), osteopontin (OPN), and PTH (parathyroid hormone). Data was analyzed using the Statistical Package, GraphPad Prism.* Results.* We found that IL-1ß cytokine is significantly higher in patients with PD (*p* value = 0.0014). However, there are no statistically significant variances found among the two studied groups with regard to the IL-6 and TNF-*α* cytokines levels. We also found that levels of PTH are decreased in the PD subjects than the age-matched controls (*p* value= 0.003). Also, the bone matrix glycoproteins, including osteoprotegerin (OPG) and osteopontin (OPN), are significantly upregulated (*p* value= 0.04 for OPG and p value= 0.003 for OPN), as compared to the controls.* Conclusions. *Our findings are reliable with the possibility that inflammatory and bone markers can be used as biomarkers in PD prognosis. However, to clarify the natural role and consequence of these markers in PD pathology, further larger cohort studies are needed.

## 1. Introduction

Parkinson's disease (PD) is a chronic, multifaceted disorder known as the second most commonly neurodegenerative disease, after Alzheimer's disease. PD is uncommon before the age of 50; however, the prevalence rises with age, affecting about 1% of the population > 60 years old. A few research studies have found that PD most commonly impacts men rather than women; however, other studies record no variations between the sexes [1].

Parkinson's disease is neuropathologically recognized via the inclusions of Lewy bodies and Lewy neurites containing *α*-synuclein (*α*-syn) [2]. While the etiology of PD is multifactorial, the protein *α*-syn is a critical component to the disease's pathogenesis. However, the mechanism that causes toxicity leading to neuronal death through *α*-syn malfunction remains unknown.

Parkinson disease is mostly characterized through the gradually death of dopaminergic nerves within the substantia nigra pars compacta (SNpc), subsequently principal of dopamine deficit on the striatum [3]. Clinically, patients with PD show a variety of symptoms, but the clinical hallmarks are resting tremor, postural imbalance, akinesia, bradykinesia, and rigidity, while nonmotor symptoms consist of cognitive impairment, depression, autonomic disorder, dementia, and visual hallucination [4].

Neuroinflammation, known in the pathogenesis of PD, has been suggested to play a crucial role in loosing neuronal in dopaminergic cells within the substantia nigra and then influencing the development of PD symptoms [1, 2]. Several studies have confirmed an increase in the peripheral cytokines, such as TNF and IL-6, in patients with PD when compared with control subjects [5, 6]. Moreover, a study found that fatigued PD patients showed elevated levels in IL-6 serum concentration when compared with nonfatigued patients. These outcomes suggest that IL-6 might also have a role that may cause fatigue in cases with PD [7].

Osteopontin (OPN) was revealed to be elaborate in inflammatory and degenerative mechanisms of the neurones (Carecchio et al., 2011). OPN plays a critical role in PD due to its anti-inflammatory and antiapoptotic properties and its role in regulating iNOS transcription, reactive oxygen species production, and cytokines levels [31–33]. In addition, it is been found that OPN sera and cerebrospinal fluid (CSF) amounts are greater in PD patients than controls, with CSF extent positively linked with concomitant dementia (Maetzler et al., 2007).

Parkinson disease is a complicated heterogeneous disease that requires multimodal biomarker technique to track and control the disease progression and analysis. Within the Saudi population, the association between these biomarkers and PD was not previously studied. Understanding the pathophysiological mechanisms of PD would lead to the development of effective therapies. Therefore, it is very crucial to study and discover a likely relationship between PD pathogenesis and serum concentrations of these biomarkers.

## 2. Methodology

This project was approved by the King Fahad Medical City (KFMC), IRB Committee. Seventeen PD patients were enrolled from the Outpatient Neurology Clinic at KFMC, Riyadh. Additionally, 9 cases were recruited from King Abdulaziz University Hospital (KAUH) in Jeddah. A formal written consent form was provided to all included subjects to read and sign prior to the study.

### 2.1. Subjects

Seventeen patients were included from KFMC in this study and 9 patients from KAUH Applicable demographic and clinical data were collected from each patient's medical record. Additionally, the control group included 24 Saudi individuals, which were matched with PD patients in regard to their age and sex (IRB number 16-450, IRB registration number with KACST, KSA H-01-R-012, IRB registration number with PHRP/NIH/USA = IRB00010471, Approval number federal wide assurance NIH, USA = FWA00018774).

### 2.2. Vitamin D, Calcium, and Phosphate Assay

Vitamin D was evaluated by determining 25-dihydroxyvitamin D (25-OH D) levels using ELISA with a commercially available radioimmunoassay (R&D systems, refRDKAP1971). 150 *μ*L of Incubation Buffer was added to microplate. Then, the plate was incubated for 2 hours at room temperature (RT), on a plate shaker (400 rpm). After that, the plate was rinsed 3 times by washing solution. Then, 200 *μ*L of the Working HRP conjugate solution was added to plate wells and then incubated for 30 minutes at RT on a plate shaker (400 rpm). Again, the plate was rinsed 3 times by washing solution. Later, 100 *μ*L of the chromogenic solution was added to each well within 15 minutes following the washing step. Then, the plate was incubated for 15 minutes at RT, on a plate shaker (400 rpm). Finally, color development was stopped via addition of 100 *μ*L of Stop media into separate well; then absorbance was calculated at 450 nm within 1 hour.

### 2.3. Cytokines Multiplex Assay

Human bone magnetic bead panel, cytokines multiplex assay from Milliplex (Cat No HBNMAG-51K), was used to assess the levels of IL-1*β*, IL-6, TNF-*α*, osteoprotegerin, osteopontin, and PTH. Frozen serum samples, from PD versus non-PD subjects, were assessed for all the above parameters in duplicate at one time by using a single plate. The procedure was done according to the assay protocols provided by the manufacturer. Luminex 200 machine and Milliplex Analyst software were used for data analysis, at the Neuroscience Unit in KAUH.

The kit uses a 96-well format, containing a lyophilized standard cocktail and 2 quality controls that can measure up to 38 serum samples in duplicate. Multidimensional fatigue inventory (MFI) measurements were obtained, and data was analyzed accordingly for high sensitivity, consistency, and reproducibility.

In summary, 25 *μ*g of serum (1:2 diluted) was incubated with antibody-conjugated magnetic beads for overnight at four-degree temperature inside the fridge. Bead-complexes after rinsed were kept with 50 *μ*L biotinylated detection antibody for half an hour on a plate shaker at room temperature. After that, they were incubated with 50 *μ*L streptavidin-phycoerythrin for half an hour on a plate shaker at (20-25°C). After washing 3 times 100 *μ*L of Sheath Fluid was added to all wells. Bead-complexes were then read on Run plate on Luminex® 200TM and analyzed by MAGPIX® with xPONENT® software.

### 2.4. Statistical Analysis

GraphPad Prism 7 software was used to perform an unpaired t-test to compare between the PD and control groups using all the investigated biomarkers.

## 3. Results

### 3.1. Patients' Characteristics

Information on the sociodemographic characteristics and initial laboratory parameters of vitamin D (25-Hydroxyvitamin D), calcium, and phosphate was attained via closed question form (Table 1).

### 3.2. Status of the Inflammation in the PD

**Figure 1:**
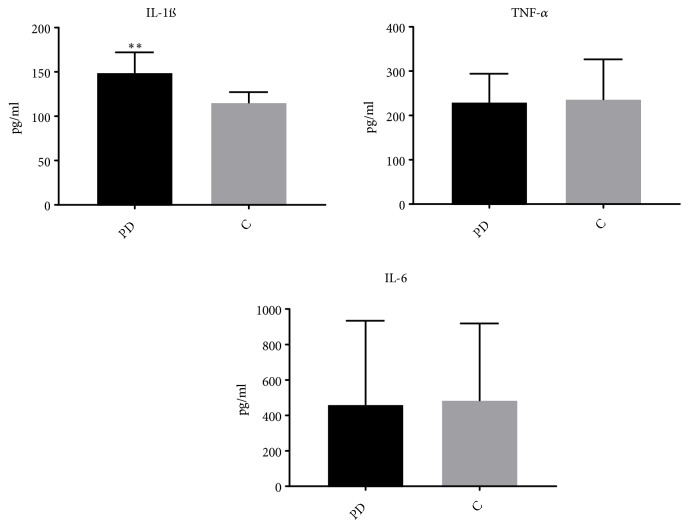
*IL-1ß cytokine is upregulated in the PD*. Results of inflammatory cytokines IL-1ß, IL-6, and TNF-*α* are differentiated between the PD and control (C) subjects. Data are expressed as mean ± standard error of the mean. An unpaired t-test was used for comparison (n = 50); *∗∗p* < 0.001.

In comparison to the controls, IL-1ß cytokines were significantly higher in patients with PD (p value = 0.0014), (Figure 1). However, the IL-6 and TNF-*α* cytokines did not significantly differ between the two studied groups.

### 3.3. Imbalance of Bone Matrix Glycoproteins and PTH in PD

**Figure 2:**
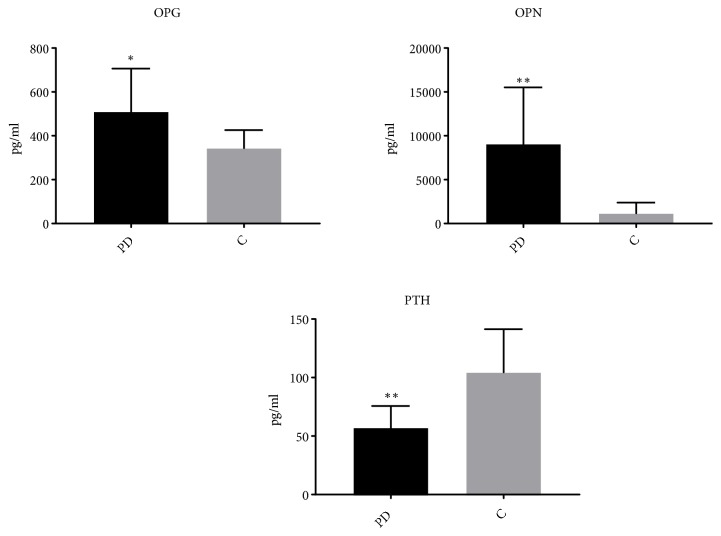
*Bone matrix glycoproteins are overshoot from PD. *Results of bone matrix glycoproteins: osteoprotegerin (OPG) and osteopontin (OPN) and parathyroid hormone (PTH) are differentiated between the PD and control (C) subjects. Data are expressed as mean ± standard error of the mean. An unpaired t-test was used for comparison (n =50); *∗∗p* < 0.05 and *∗∗p* < 0.001.

Despite the lower levels of PTH produced in the PD subjects than the age-matched controls (p value= 0.003), the bone matrix glycoproteins, including osteoprotegerin (OPG) and osteopontin (OPN), were significantly upregulated (p value= 0.04 for OPG and p -value= 0.003 for OPN), as compared to the controls. The results are depicted in Figure 2.

## 4. Discussion

Biomarkers are commonly used to serve as a predictor tool in pathogenic processes and normal biological function to expand our understanding and treatment of complex diseases. The function of microglia in the pathology of PD had been established twenty years ago from a postmortem study when scientists found T-lymphocytes activation and microglia dysfunction in the SNpc of PD subjects [8]. Thus far, various investigations have exhibited the connection of inflammatory microglia in the pathology of PD.

Cytokines display a functioning role in several diseases and conditions, which are inflammation host responses to infection, injury, sepsis, and malignant growth. Cytokines are nonstructural proteins that contain low molecular weights running from 8,000 to 40,000 Da [9]. Various immune and nonimmune cells (e.g., macrophages, T-lymphocytes, Schwann cells, and fibroblasts) are recognized to create cytokines that are essential in cell signalling. Cytokines, including interleukins, interferon, and chemokines, are in charge of inciting numerous biological impacts, for example, inflammatory responses, inhibition or stimulation of cell development and differentiation, cytotoxicity/apoptosis, and antiviral action [10]. Additionally, cytokines are imperative in the inflammatory or anti-inflammatory processes relying upon the need of the host's biological status [10].

There are two types of cytokines based on their actions: (1) proinflammatory cytokines (IL-1, TNF) that are engaged with starting inflammation and (2) anti-inflammatory cytokines (IL-4, IL-10, IL-13, and TGF) which control the proinflammatory cytokines' action. Moreover, cytokines have a vital role in both inflammatory and anti-inflammatory forms in numerous neurological diseases [10–12]. Various cytokines are secreted by neurons or glia, thereby changing cytokines concentration in the brain, blood, and cerebrospinal fluid (CSF) of patients with PD [11].

Our results as shown in Figure 1 confirmed previously published data that the Th1 cytokines (TNF-*α*,) and some proinflammatory cytokines (such as IL-6) are similar in the serum of PD patients. Moreover, we found a significant increase in IL-1*β* serum concentration in PD subjects.

Various investigations have reported that IL-1 has a primary function in the inflammation process, together with the dynamic role in the development of a multifaceted hormonal and cell inflammatory course [13]. Although abnormal increase in IL-1 level was linked with neuronal degeneration, elevated levels of IL-1 concentrations have been seen in CSF and brain parenchyma of humans and rodents after brain trauma [11, 13, 14].

Additionally, up expression of IL-1 is the fundamental element associated in the initiation of the inflammatory course, which consequently initiates a vicious cycle of various reactions that subsequently lead to neuronal loss. In the present study, serum concentration of IL-1*β* was remarkably higher in patients with PD in comparison to controls as indicated in Figure 1. Our finding is in agreement with previous investigations that associated increase in IL-1*β* serum concentration with PD diagnosis.

Moreover, it has been reported that IL-6 may be correlated with inflammation process or neuronal survival in the brain which may affect the neurodegeneration process in PD [12]. Although we did not find a significant difference between IL-6 concentrations in our patients with PD and control cases Figure 1, it is important to know that IL-6 neuroimmune dysfunction was linked with central nervous system (CNS) inflammation. This is merely because it was documented in the CSF and postmortem brain of PD patients and, it is not likely to determine the origin of cytokines when serum cytokines levels are measured.

On the other hand, TNF-*α* is a member of the peptide ligands family which contributes the stimulation of a set of structurally interassociated receptors [15]. TNF-*α* is a protein of 17 kDa molecular weight, that consists of a nonglycosylated protein of 157 amino acids. The TNF-*α* is biological response to the stimulation via two structurally different receptors. Both types of receptors are transmembrane glycoproteins that have more than one cysteine-rich which repeats in the extracellular N-terminal domains [16]. In the peripheral system, the activated macrophages and T-lymphocytes are mainly accountable for the creation of pro-TNF (molecular weight 26 kDa) protein [15]. TNF is expressed on the plasma membrane and then processed via matrix metalloproteinase, leading to cleavage of the extracellular domain and finally yielding 17 kDa soluble structure of TNF [17]. Keep in mind that a range of pathological approaches including inflammation process, ischemia, and traumatic damage can lead to the mediation of microglia and astrocytes in order to introduce TNF in the CNS [18].

TNF is known as a circulating element leading to tumor necrosis. Although studies have validated that TNF has crucial and numerous function in the pathological development of several chronic diseases such as neurodegenerative disorders [18]; typically, in the brain TNF performs a critical position structurally and functionally such as normal behavior, sleep, and synaptic plasticity [19]. In contrast, activation of the microglia in the CNS causes elevated TNF-*α* expression which subsequently stimulates NFK-*α* function [20]. iNOS is needed in the production of NO as well as peroxynitrite, known as NO-derived reactive nitrogen species [14]. Therefore, upregulation of large reactive molecules results in the production of lipid peroxidation, tyrosine nitrosylation, and oxidative damage to DNA ensuing in neurodegeneration [14]. Subsequently, this neural damage may play a possible role in PD pathology [14, 20].

A study has found that TNF levels were elevated in a cohort of Japanese early-onset PD patients in comparison to late-onset of PD and control subjects [21].

A notable number of studies which have been done in peripheral inflammatory/immune markers suggested the hypothesis of inflammation involved in PD [22]. Furthermore, research of cytokines in plasma and serum was found to raise levels of proinflammatory cytokines such as TNF-*α* and its soluble receptors sTNFR1 and IL-1*β* in PD cases in comparison with matched controls [22, 23].

In this study, levels of TNF-*α* did not change between PD patients and healthy controls Figure 1. Therefore, our findings can be explained by the heterogeneity of PD pathophysiology. Although IL-6 plasma level was prospectively related to an elevated risk of developing PD [24]; several studies have failed to point out noteworthy changes in cytokines (IL1-*α*, IL-6, and TNF-*α*) concentration in PD.

## 5. Conclusions

The interpretation of the findings of this study should take into account many limitations, such as medications used by PD patients, disease severity, and small sample size. In addition, the cytokine methodology assessment may differ which directly affects result's sensitivity. In conclusion, our findings demonstrated that serum and IL-6, IL-IB, and TNF could be used as promising applicable biomarkers of PD inflammation. However, in this study, we did not find a significant impact of IL-6 and TNF-*α* concentrations on PD patients. Our results revealed that inflammatory mechanisms are an essential factor in PD pathophysiology.

## Figures and Tables

**Table 1:** Sociodemographic characteristics of the subjects.

Variables	PD cases (n = 26)	Control Cases (n = 24)	*P-value*
Age (years)	60.35 ± 11.5	60.22 ± 9.2	*0.9769 NS*

Sex	20 M / 6 F	18 M / 6 F	

Duration of disease (years)	6.23 ± 4.5	NA	

Vitamin D Supplement use			
Yes	16	6	
No	10	18	

Sun exposure			
Yes	3	4	
No	23	20	

Family history of PD (first degree)			
Yes	9	0	
No	17	24	

Laboratory parameters			
Vitamin D (25-Hydroxyvitamin D)(nmol∖l)	58.7 ± 12.8	156.9 ± 13.7	0.0001
Calcium levels (mg∖dl)	8 ± 0.0	9.2 ± 0.22	0.0253
Phosphorus levels (mg∖dl)	2.6 ± 0.1	3.4 ± 0.29	0.0561

M, male; F, female; NA, not applicable; NS, not significant. Data are expressed as mean ± standard deviation.

## Data Availability

The data used to support the findings of this study were included within the article.
