# Erratum to “Assessment of the Levels of Level of Biomarkers of Bone Matrix Glycoproteins and Inflammatory Cytokines from Saudi Parkinson Patients”

**DOI:** 10.1155/2020/7254325

**Published:** 2020-09-26

**Authors:** Aziza Alrafiah, Ebtisam Al-Ofi, Mona Talib Obaid, Nimah Alsomali

**Affiliations:** ^1^Department of Medical Laboratory Technology, Faculty of Applied Medical Sciences, King AbdulAziz University, Jeddah, Saudi Arabia; ^2^Department of Physiology, Faculty of Medicine, King Abdulaziz University, Jeddah, Saudi Arabia; ^3^National Neuroscience Institute, King Fahad Medical City, Riyadh, Saudi Arabia

In the article titled “Assessment of the Levels of Level of Biomarkers of Bone Matrix Glycoproteins and Inflammatory Cytokines from Saudi Parkinson Patients” [1], multiple references were omitted in error. The references are shown below and listed as [2, 3].

These should supplement the following sentences in the article text:

Osteopontin (OPN) was revealed to be elaborate in inflammatory and degenerative mechanisms of the neurons [2]. OPN plays a critical role in PD due to its anti-inflammatory and antiapoptotic properties and its role in regulating iNOS transcription, reactive oxygen species production, and cytokines levels (9–10). In addition, it has been found that OPN sera and cerebrospinal fluid (CSF) amounts are greater in PD patients than controls, with CSF extent positively linked with concomitant dementia [3].

The error was introduced during the production process of the article, and Hindawi apologises for causing this error in the article.
